# Treacher-Collins Syndrome–A Challenge For Anaesthesiologists

**Published:** 2009-08

**Authors:** Leena Goel, Santosh Kumar Bennur, Shweta Jambhale

**Affiliations:** 1 Lecturer; 2,3 Senior Resident

**Keywords:** Treacher-Collins syndrome, anaesthesia management

## Abstract

**Summary:**

Treacher-Collins syndrome is a rare congenital disease known to be associated with a difficult airway and presents some of the most hazardous and difficult challenges that anaesthetists may encounter within the entire practice of paediatric anesthesia. Successful anaesthetic management in a case of Treacher-Collins syndrome posted for cleft palate repair is presented in this report.

## Introduction

Treacher-Collins syndrome is also referred to as mandibulo–facial dysostosis is a highly complex disease process. It is a congenital malformation of first and second bronchial arch, inherited as autosomal dominant trait.^1^ The basic etiology is obscure. Ida Maan in 1943 mentioned that a disturbance in division and development of the mesodermal bone tissue at the time of the fifth week offo etal life probably initiates this syndrome.^2^ lt is seen mostly among the poorer class of people.

The syndrome consists of congenital and familial deformities of the ear, eyes, maxilla and mandible. It is often associated with deafness due to meatal atresia and malformation of the middle and inner ear.^3^ Cleft lip and palate is present in up to 35% ofpatients, 34-30% have congenital palatopharyngeal incompetence, and are associated with other malformations of cardiovascular system. During the post operative period, pharyngeal and laryngeal edema may develop. Even respiratory distress and sudden death has been reported.^4^

## Case Report

**Fig 1:**
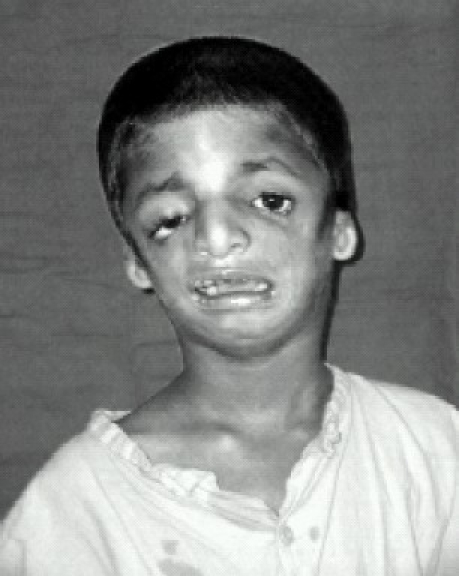
Front view; Treacher- collins syndrome patient showing hypoplasia of facial bones, down sloping palpabral fissures, coloboma of the lower eyelids with scanty lower eye lashes.

**Fig 2:**
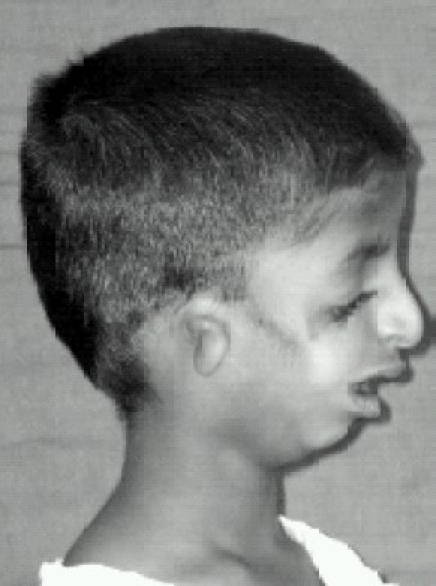
Lateral view; showing the deformity of ear (microtia) and retrognathis

Aboy aged 5 years weighing 10kg, diagnosed as Treacher-Collins syndrome was presented for surgery for cleft palate repair. On evaluation the patient was found to have hypoplasia of facial bones (mandible, maxilla and cheek), down sloping palpebral fissures, coloboma of the lower eyelids, scanty lower eye lashes, microtia with hearing loss and micrognathia and retrognathia (Fig 1, 2). On airway assessment mouth opening was adequate and he had a Mallampatti class 4 airway. Neck movements were normal and spine was normal. Preoperative blood investigations showed Hb 12gm%, BUN, creatinine and electrolytes were normal. Chest X-ray and ECG were normal. In view of the difficult airway, relatives were informed that the technique might fail and tracheostomy consent was taken. A trolley for difficult airway was kept ready including LMA, retrograde intubation set, tracheostomy set and emergency cricothyroidotomy set.

The patient was kept nil by mouth for six hours. Age was a limitation for awake intubation. We planned to go for smooth induction with a deeper plane of anesthesia, avoiding hypoventilation and trauma to the airway. The patient was premeditated with atropine 0.2mg IV given to reduce the secretions. Sedatives were avoided as we anticipated a difficult airway. Dexam-ethasone 0.2mg.kg^-1^ IV was given. The patient was preoxygenated with 100% oxygen for five minutes. Induction was done with IV propofol 2mg.kg^-1^ with incremental dose of sevoflurane. Initially mask ventilation seemed to be difficult due to a poor mask fit but improved to some extent after an orophrayngeal airway insertion and gauze packing of the space between the mask and the cheek. But even with this we could not ventilate adequately. Then one assistant was asked to lift forward both the angles of the jaw, and only then the patient could be ventilated. After induction, a gentle laryngoscopy showed a Class IV of glottis visualization as per Cormack Lehane classification.

**Fig 3:**
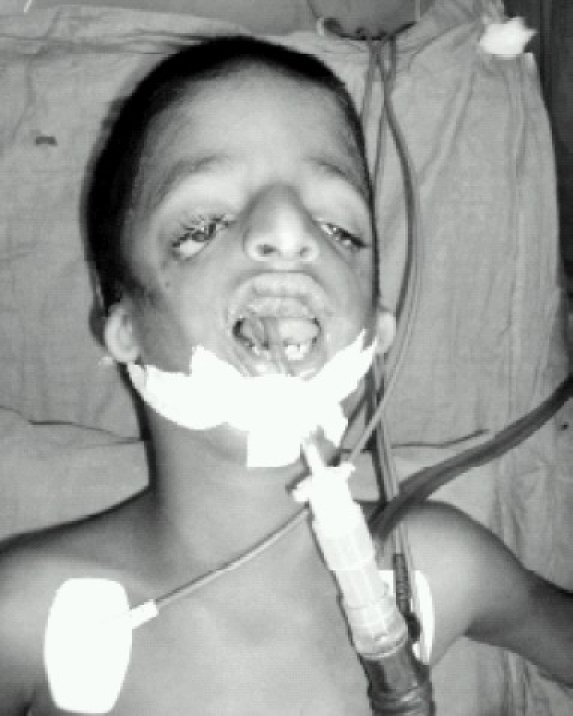
Intubation with a no.4 uncuffed RAES endotracheal tube

Now taking the patient deeper, another assistant was asked to give a very good backward upward right-ward pressure (BURP). With this maneuver laryngoscopy showed a Cormack Lehane of glottis visualization as Class III. Now we were able to intubate with a No.4 uncuffed RAES endotracheal tube with the help of an appropriate sized stylet (Fig. 3). Later the tube was secured properly and the patient was handed over to the surgeons.

Further anaesthesia was maintained with N_2_O+ O_2_+ sevoflurane and pancuronium with a supplementation of fentanyl 2μg.kg^-1^ IV for analgesia. The patient was monitored with pulse oximetry, EtCO2, NIBP, ECG, and precordial stethoscope throughout the surgical period which lasted for about two hours. The oxygen saturation, heart rate, BP, EtCO2 were monitored and maintained. At the end of the surgery, the patient was reversed with neostigmine 0.05mg.kg^-1^ and atropine 0.02mg.kg^-1^. A smooth extubation was done once the child was fully awake. The patient was kept under observation and the postoperative period was uneventful. The patient was discharged after 7 days without any complication.

## Discussion

Patients with Treacher-Collins syndrome present a serious problem to anaesthetists in maintaining their airway, as upper airway obstruction and difficult tracheal intubation due to severe facial deformity make such a task difficult. Because of retrognathia, the airway management of these patients is often challenging.^5^,^6^

The cause for difficult intubation in such cases is due to relative macroglossia as a consequence of skeletal abnormalities. This reduces the space available for manipulation and insertion of the endotracheal tube (ETT). The associated abnormalities may be limited mouth opening, reduced extension of the head on the neck, hypoplastic mandible and limited forward movement of hyoid. Often multiple mechanisms may be present in an individual case.^7^‐^10^

Treacher-Collins syndrome is caused by a defective protein called treache.^11^ More than half of the cases are thought to be due to new mutations. Because there is no family history of the disease, the condition may greatly vary in severity from generation to generation. Treacher Collins syndrome is a congenital deformity mainly of the face, and frequently of other parts of body. It is characterized by hypoplasia of the facial bones, especially the zygoma and mandible. Facial clefting causes the hypoplastic appearance, with possible deformities of the ear, orbital, midface and lowerjaw regions. The clinical appearance is a result of zygoma (malar bone) failing to fuse with the maxilla, frontal, and temporal bones.^12^

### The main signs of syndrome,^2^

1-Skeleton-(a) Skull-square forehead, flattening of the occipito-parietal region, lack of angulations in the naso–frontal region and long occipito-frontal diameter, asymmetry of the skull caused by maldevelopment of the internal structure of the skull, such as petrosal bone.

b) Facial bones- Malars under developed with small ridges, lack of zygomatic arch, small antra and in some cases antra disproportionally large to the small malar bone, hypertrophic nasal septum, hypertrophic maxillary arch, frequently highly arched palate, may be associated with cleft palate, malgrowing teeth, mandible hypoplastic, retruded with angle of jaw obtuse which frequently gives an open bite.

c) Others- May have in some cases, fused cervical vertebrae, abnormal costo-sternal structure, such as pectus excavates, long metacarpal and metatarsal bones.

2-Eye-Narrow interpalpebral spaces, most frequently running obliquely laterally, but occasionally medially. Notching on the outer portion of the lower eyelid mostly, and occasionally on the upper eyelid. The eyelashes are absent or growing irregularly in two or three rows. Absence of lachrymal glands, lateral deviation or protrusion of the eyeball, congenital ptosis.

3-Ears- Congenital absence of the external ear, absence or occlusion of auditory meatus, deformity of the ear, with partial or complete deafness.

4-Mouth-Mostly there is macrostomia and in some cases microstomia, occasionally undeveloped tongue.

5-Atresia of pharyngeal ring.

6-Cheeks-Blind fistulae on the cheeks and rarely “tongue–shaped” growth of hair on the cheeks on the lateral side of face.

7-Nose-In the majority of cases it is big with a very short columella, narrow nares and small lateral cartilages.

8-Associated deformities-Hypermobility of the phalanges.

9-Mental development of children with this syndrome-Typically grows to become normal functioning adults of normal intelligence.

Our patient was a classical case of Treacher-Collins syndrome with almost all the features with a significant airway distortion, because of which we had expected difficulty in maintaining airway as well as difficult tracheal intubation.

Various techniques have been described in management of such patients. These includes; direct laryngoscopy, intubation with a flexible fiberoptic broncho-scope, light wand, laryngeal mask airway, retrograde intubation technique and tracheotomy can also be employed.^13^

In awake, fiberoptic intubation for a recognized difficult airway in the pediatric population is challenging, if not impossible secondary to the lackof cooperation by the sedated child. Some alternatives to direct laryngoscopy include blind light wand technique, blind nasal intubation, and oral or nasal fiberoptic intubation; however these techniques are often more difficult in the pediatric population.

These difficulties might be avoided by first attempting intubation through a Laryngeal Mask ay (LMA) rather than direct laryngoscopy. However, the greatest challenge encountered when intubating through an LMA is how to remove the LMA without dislodging the ETT from the trachea. This difficulty is unique to the pediatric anaesthesiologist, because the lengths of an ageappropriate pediatric ETT and LMA are similar, and the proximal end of the ETT tends to disappear in to the LMA once the ETT has passed through the vocal cords. This makes it difficult to safely remove the LMA without dislodging the ETT. One can circumvent this problem by extending the length of the pediatric ETT. However, the paediatric patients with Treacher-Collins syndrome have the posteriorly protruded tongue which displaces the LMA, makes the glottis move considerably anterior and interfere with the attempts to enter the trachea with a bougie. Further downward displacement of the epiglottis can also impair the intubation technique through LMA. The same is true for light wandguided intubation.^5^,^14^,^15^

Though the success rate with retro grade tracheal intubation is higher, it is more uncomfortable and atraumatic experience to the awake patient. This is also an uneasy approach in pediatric patients as it needs a lot of understanding and cooperation from patient.

Comparatively our conventional approach with additional maneuvers seems abetter option, and though it is less predictable, it is without complications. In our hospital, as the above facilities were not available, we planned to go for smooth induction with adeeper plane of anaesthesia with direct laryngoscopy.

The three most important modifications we used in this technique were:

- Since we had expected difficult ventilation and intubation in this patient, we preferred to keep the patient spontaneously breathing with no muscle relaxation but in a deeper plane of anaesthesia to give us ample time to attempt intubation.
- The forward lift of both the angles of the mandible by an assistant which to greater extent overcome the main cause of difficult ventilation in Treacher-Collins syndrome, the retrognathia.
- And finally intubation was facilitated by a very good backward upward and rightward pressure (BURP) by an assistant which makes Cormack Lehane of glottis visualization as Class III.

Anesthesiology is a field of challenges, especially when you encounter difficult to ventilate and difficult to intubate scenario. Hence every pediatric anaesthe-siologist should be well prepared with the various techniques of the difficult airway algorithm before venturing into a case. This case of Treacher-Collins syndrome illustrates how a modified conventional approach can still be a very good and gold standard approach when other newer techniques are not available.
